# Association between COMISA and cardiovascular disease: Evidence from the TURKAPNE registry

**DOI:** 10.1371/journal.pone.0354810

**Published:** 2026-07-31

**Authors:** Yüksel Peker, Aylin Pihtili, Esen Kiyan, Mehmet Sezai Tasbakan, Semih Arbatli, Senay Aydin, Aykut Cilli, Neşe Dursunoglu, Burcu Baran, Özen K Basoglu, on behalf of the Turkapne Study Group

**Affiliations:** 1 Department of Pulmonary Medicine, Koc University School of Medicine, Istanbul, Türkiye; 2 Department of Molecular and Clinical Medicine, Institute of Medicine, University of Gothenburg, Sahlgrenska Academy, Gothenburg, Sweden; 3 Department of Clinical Sciences, Respiratory Medicine and Allergology, Lund University School of Medicine, Lund, Sweden; 4 Division of Pulmonary, Allergy, and Critical Care Medicine, University of Pittsburgh School of Medicine, Pittsburgh, Pennsylvania, United States of America; 5 Koc University Research Center for Translational Medicine (KUTTAM), Istanbul, Türkiye; 6 Department of Pulmonary Medicine, Istanbul University School of Medicine, Istanbul, Türkiye; 7 Department of Pulmonary Medicine, Ege University School of Medicine, Izmir, Türkiye; 8 Department of Neurology, Yedikule Chest Diseases and Thoracic Surgery Education and Research Hospital, Istanbul, Türkiye; 9 Department of Pulmonary Medicine, Akdeniz University School of Medicine, Antalya, Türkiye; 10 Department of Pulmonary Medicine, Pamukkale University School of Medicine, Denizli, Türkiye; 11 Department of Pulmonary Medicine, Erciyes University School of Medicine, Kayseri, Türkiye; Rutgers Robert Wood Johnson Medical SchoolM, UNITED STATES OF AMERICA

## Abstract

**Background:**

Comorbid insomnia and obstructive sleep apnea (COMISA) is increasingly recognized as a distinct clinical entity, yet its relationship with cardiovascular disease (CVD) remains incompletely understood. We aimed to investigate the association between COMISA and CVD in a large clinical sleep cohort.

**Methods:**

Using data from the TURKAPNE registry, we analyzed 12,715 adults referred for sleep evaluation and classified participants into four mutually exclusive sleep phenotypes: neither OSA nor insomnia (n = 910), insomnia only (n = 388), OSA only (n = 8,142), and COMISA (n = 3,275). The prevalence of CVD was compared across groups. Multivariable logistic regression models were used to examine associations between COMISA and CVD after adjustment for age, sex, body mass index, education level, smoking status, airway disease, diabetes mellitus, and psychiatric comorbidity.

**Results:**

The prevalence of COMISA was higher among individuals with CVD compared with those without CVD (31.6% vs. 22.7%; p < 0.001). In contrast, the distribution of insomnia alone and OSA alone showed only minor differences by CVD status. In multivariable analysis, CVD was independently associated with COMISA (odds ratio 1.108, 95% confidence interval 1.006–1.220; p = 0.038), although the magnitude of this association was modest. Female sex, diabetes, airway disease, psychiatric disease, lower educational status, and current smoking were also independently associated with COMISA.

**Conclusion:**

In this large registry-based cohort, COMISA was more prevalent among individuals with CVD, supporting the concept that it represents a clinically distinct phenotype with an increased cardiometabolic burden. However, the modest effect size suggests that COMISA is influenced by a broader interplay of behavioral and metabolic factors.

## Introduction

Obstructive sleep apnea (OSA) and insomnia are among the most common sleep disorders in adults and frequently coexist in clinical practice. This overlap is referred to as comorbid insomnia and sleep apnea (COMISA) [1]. OSA is characterized by recurrent upper airway obstruction during sleep, leading to intermittent hypoxemia, sleep fragmentation, intrathoracic pressure swings, and sympathetic activation [2]. These mechanisms contribute to hypertension, metabolic dysfunction, endothelial injury, and adverse cardiovascular outcomes [3,4]. Insomnia, on the other hand, is associated with hyperarousal, hypothalamic–pituitary–adrenal axis activation, increased sympathetic tone, inflammation, and impaired cardiometabolic regulation. When both conditions coexist, these pathophysiological pathways may interact and produce an additive or synergistic cardiovascular burden rather than representing a simple coexistence of two independent sleep disorders [5,6].

COMISA is increasingly recognized as a distinct clinical phenotype within the OSA spectrum. Previous studies have shown that approximately 30–50% of patients with OSA report insomnia symptoms, while a substantial proportion of patients with insomnia also meet criteria for OSA [3]. Patients with COMISA often have more severe sleep fragmentation, prolonged nocturnal awakenings, impaired daytime functioning, fatigue, and psychological distress compared with those with OSA alone. Importantly, this phenotype may also be associated with a higher burden of cardiovascular and metabolic disease, including hypertension, coronary artery disease, heart failure, stroke, and increased mortality [1,7].

Recent epidemiological and clinical data support the association between COMISA and cardiovascular disease (CVD). In a large real-world cohort using the TriNetX U.S. Collaborative Network, Lu et al. compared 165,522 patients with COMISA and 165,522 patients with OSA alone after propensity score matching. COMISA was associated with increased 10-year risks of cerebrovascular disease, arrhythmias, inflammatory and ischemic heart disease, and thrombotic disorders compared with OSA alone, suggesting that insomnia comorbidity may add clinically relevant cardiocerebrovascular risk among patients with OSA [3]. Similarly, in a nationwide cohort of post-9/11 U.S. Veterans, COMISA conferred the highest risk of incident hypertension and other CVDs, with more than a twofold increase in hypertension risk and more than a threefold increase in other CVD risk after adjustment for demographic, behavioral, and clinical factors [8].

Hypertension appears to be one of the most consistent cardiovascular outcomes associated with COMISA. In the SCAPIS Gothenburg population-based cohort, Kobayashi Frisk et al. reported that uncontrolled hypertension was more frequent in individuals with COMISA than in those with OSA alone, insomnia alone, or neither disorder. The association remained significant after adjustment for anthropometric factors, lifestyle, comorbidities, sleepiness, and nocturnal hypoxic exposure, with time spent below 90% oxygen saturation acting as a significant mediator [9]. In another recent study of hypertensive OSA patients, Durak et al. found that COMISA was independently associated with resistant hypertension, even after accounting for age, body mass index, and apnea–hypopnea index [4]. These findings suggest that COMISA may identify a subgroup of OSA patients with particularly unfavorable blood pressure control, independent of conventional markers of OSA severity.

The prognostic importance of COMISA may also depend on specific insomnia phenotypes. In the Penn State Adult Cohort, Athanasiou et al. showed that the highest risk of incident hypertension was observed in individuals with COMISA combined with objective short sleep duration [10]. In contrast, insomnia with normal objective sleep duration, either alone or in combination with OSA, was not significantly associated with incident hypertension. These findings suggest that objective short sleep duration may help identify a biologically more severe COMISA phenotype with greater cardiovascular vulnerability. Supporting this concept, Pejovic et al. demonstrated that among adults with mild-to-moderate OSA and insomnia symptoms, the insomnia with short sleep duration phenotype was associated with higher systolic and diastolic blood pressure, increased hypertension risk, elevated IL-6 levels, and greater insulin resistance compared with insomnia with normal sleep duration [11].

Earlier studies also support a possible synergistic effect of COMISA on cardiovascular risk in high-risk populations. Draelants et al. found that, among hypertensive subjects, COMISA—but not OSA or insomnia alone—was independently associated with high 10-year cardiovascular disease risk assessed by the Framingham Risk Score [6]. Similarly, Hein et al. reported that COMISA was associated with high 10-year cardiovascular risk in patients with major depression, whereas insomnia disorder or OSA alone were not independently associated with this outcome [12]. In patients with type 2 diabetes, Hein et al. also showed that COMISA, rather than either component alone, was associated with higher cardiovascular disease risk, further supporting the concept that COMISA may exert a negative synergistic effect in metabolically vulnerable populations [13].

Despite growing evidence, several important gaps remain. Existing studies vary in their definitions of insomnia, OSA, COMISA, and cardiovascular outcomes. Some studies rely on diagnostic codes, whereas others use questionnaire-based insomnia definitions, polysomnography, or home sleep apnea testing. Moreover, many studies are cross-sectional or based on selected clinical populations, making it difficult to establish temporality or determine whether COMISA is associated with cardiovascular outcomes beyond traditional risk factors and OSA severity. Nevertheless, accumulating evidence suggests that COMISA is associated with a higher cardiovascular burden than OSA or insomnia alone and may represent a clinically meaningful target for cardiovascular risk stratification.

To further address this evidence gap, we analyzed baseline data from the Turkish Sleep Apnea Database (TURKAPNE), a nationwide, multicenter, observational registry designed to systematically capture clinical characteristics, sleep study data, and comorbidities in adults referred for sleep evaluation across Türkiye [14,15]. Using this real-world registry, we evaluated the prevalence of CVD across four mutually exclusive sleep disorder phenotypes—neither OSA nor insomnia, insomnia only, OSA only, and COMISA—and hypothesized that COMISA would be associated with a higher prevalent CVD burden independent of established metabolic, respiratory, and psychiatric comorbidities

## Methods

### Study design and population

This study was based on data from the ongoing Turkish Sleep Apnea Database (TURKAPNE), a nationwide, multicenter, cross-sectional observational registry study. The rationale, design, and methodological framework of the TURKAPNE registry have been described previously [14,15]. In brief, participating centers prospectively enrol consecutive patients undergoing sleep assessment according to standard clinical practice, with harmonised data collection and predefined variable definitions.

For the present analysis, we included adults aged ≥18 years who had complete information on obstructive sleep apnea (OSA) status, insomnia status, and CVD status. OSA was diagnosed based on overnight sleep studies performed according to contemporary clinical standards, while insomnia status was determined using standard clinical criteria recorded at the time of evaluation. Comprehensive details regarding polysomnographic methodology and baseline sleep characteristics of the TURKAPNE cohort have been published elsewhere and are therefore not repeated here [14,15].

The study protocol received approval from the Ethics Committee of the Marmara University Faculty of Medicine (approval no. 09.2016.311). Written informed consent was obtained from all participants prior to study participation, and all study procedures were conducted in accordance with the principles outlined in the Declaration of Helsinki. Although this study is not a clinical trial, the registry analysis was voluntarily registered for transparency purposes at ClinicalTrials.gov (identifier: NCT02784977). The first registration was posted on 03/10/2017. Participant recruitment for the current protocol was conducted between 03/10/2017 and 31/08/2025.

### Definitions of sleep phenotypes and CVD

#### Assessment of OSA, insomnia, and COMISA.

OSA was identified based on overnight polysomnography, with a diagnostic threshold defined as an apnea–hypopnea index (AHI) of ≥5 events per hour. Symptoms of insomnia were evaluated using routinely collected self-report items within the TURKAPNE registry. Participants were considered to have insomnia symptoms if they reported frequent difficulty initiating sleep (subjective sleep latency ≥30 minutes), difficulty maintaining sleep, or current use of hypnotic medication, with responses indicating occurrence “often” or “very often.”

Information on symptom frequency (≥3 nights per week), symptom duration (≥3 months), daytime functional impairment, or adequacy of sleep opportunity was not systematically recorded in the registry. Consequently, a formal diagnosis of insomnia disorder according to DSM-5-TR or ICSD-3 criteria could not be established. Accordingly, the term insomnia symptoms rather than insomnia disorder is used throughout this study.

COMISA was defined as the coexistence of OSA (AHI ≥ 5 events/hour) and self-reported insomnia symptoms, rather than a diagnosis of insomnia disorder. Participants who met criteria for neither OSA nor insomnia symptoms were classified as the reference group. It should be noted, however, that this group consisted of individuals referred for sleep evaluation and should not be interpreted as representing healthy sleepers from the general population.

#### Assessment of CVD.

CVD was defined as a composite of hypertension, coronary artery disease (CAD), congestive heart failure (CHF), atrial fibrillation and stroke based on self-reported physician diagnosed condition.

### Statistical analysis

All statistical analyses were conducted using IBM SPSS Statistics version 28.0 for Windows. Continuous variables were presented as mean ± standard deviation (SD) or, when appropriate, as median with interquartile range (25th–75th percentiles). Categorical variables were reported as frequencies and percentages. Normality of continuous data was assessed using the Shapiro–Wilk test. Comparisons between groups were performed using the Student’s t-test for continuous variables and the Chi-square test for categorical variables. A two-sided p-value of <0.05 was considered statistically significant. Associations between COMISA and CVD were evaluated using multivariable logistic regression, with results expressed as odds ratios (OR) and 95% confidence intervals (CI).

## Results

**Fig 1:**
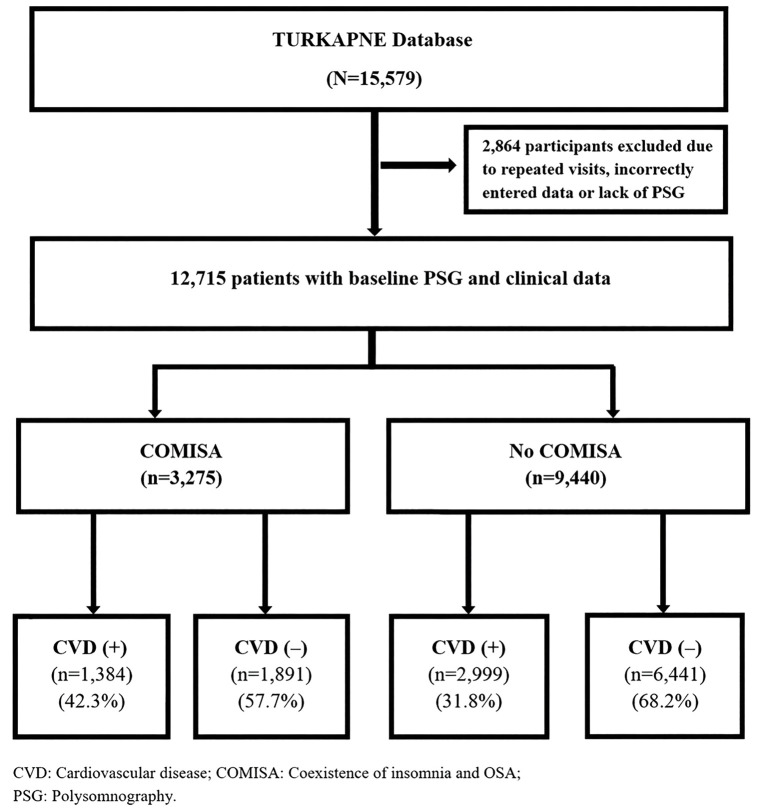
Flowchart of study population selection and distribution of cardiovascular disease (CVD) according to COMISA status in the TURKAPNE cohort.

The study population comprised 12,715 adults (the mean age: 50.2 ± 12.1 years, and the mean BMI 31.7 ± 6.1 kg/m²) referred for sleep evaluation and categorised into four mutually exclusive sleep disorder phenotypes: neither OSA nor insomnia (n = 910), insomnia only (n = 388), OSA only (n = 8,142), and COMISA (n = 3,275). Overall, 4,383 patients had CVD and 8,332 did not. As illustrated in Fig 1, among participants with COMISA (n = 3,275), CVD was present in 42.3% (n = 1,384), whereas the prevalence of CVD was 31.8% (n = 2,999) among the 9,440 participants without COMISA (p < 0.001).

**Fig 2:**
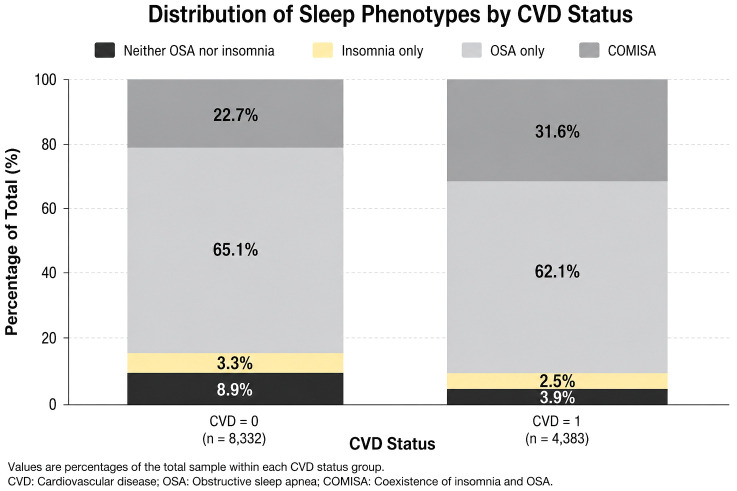
Distribution of sleep phenotypes according to cardiovascular disease (CVD) status. Percentages represent the proportion of participants within each CVD category. COMISA: coexistence of insomnia and obstructive sleep apnea.

Patients with CVD had a less favorable clinical profile than those without CVD. They were older, had higher BMI, and were more frequently obese (for all p < 0.001). They also reported a greater burden of sleep-related symptoms, including excessive daytime sleepiness, witnessed apnea, nocturia, nocturnal dyspnea, restless legs syndrome, and fatigue. In addition, diabetes mellitus, obstructive airway disease, and psychiatric comorbidity were significantly more common in the CVD group (for all p < 0.001) (Table 1). The distribution of sleep phenotypes differed significantly between groups, with COMISA being more frequent among patients with CVD than among those without CVD (p < 0.001) (Fig 2).

**Table 1:** The comparison of demographical and clinical features in the entire cohort with and without cardiovascular diseases.

	CVD (+) (n = 4383)	CVD (-) (n = 8332)	P value
**Age (years)**	55.8 ± 10.7	47.2 ± 11.7	<0.001
**Female/ male (n)**	1597/2786	2406/5926	<0.001
**BMI (kg/m2)**	33.1 ± 6.3	30.9 ± 5.8	<0.001
**Obesity (BMI > 30 kg/m2) (%)**	66.2	50.3	<0.001
**Current smoking (%)**	28.5	36.2	<0.001
**Married (%)**	86.8	82.4	<0.001
**ESS > 10 (%)**	33.6	24.3	<0.001
**No education (%)** **Primary school (%)** **Secondary school (%)** **High school (%)** **University (%)**	7.6	6.6	<0.001
	31.9	24.5	
	11.9	11.6	
	25.0	26.5	
	23.7	30.8	
**Neither insomnia nor OSA (n, %)** **Insomnia only (n, %)** **OSA only (n, %)** **COMISA (n, %)**	169, 3.9	741, 8.9	<0.001
	109, 2.5	279, 3.3	
	2721, 62.1	5421, 65.1	
	1384, 31.6	1891, 22.7	
**Witnessed apnea (%)**	43.4	31.3	<0.001
**Snoring (%)**	69.9	63.9	<0.001
**Nocturia (%)**	33.4	19.2	<0.001
**Nocturnal dyspnea (%)**	37.4	26.5	<0.001
**Restless Legs Syndrome (%)**	16.5	12.0	<0.001
**Fatigue (%)**	49.7	38.0	<0.001
**Diabetes mellitus (n, %)**	36.5	7.2	<0.001
**Obstructive airway disease (n, %)**	21.0	7.5	<0.001
**Psychiatric diseases (n, %)**	10.1	4.2	<0.001

Continuous data are presented as mean ± Standard deviation. Categorical data are presented as percentage Abbreviations: BMI: body mass index; COMISA: comorbid obstructive sleep apnea and insomnia; CVD: cardiovascular diseases; DM: diabetes mellitus; ESS: Epworth Sleepiness Score; OSA:obstructive sleep apnea.

**Fig 3:**
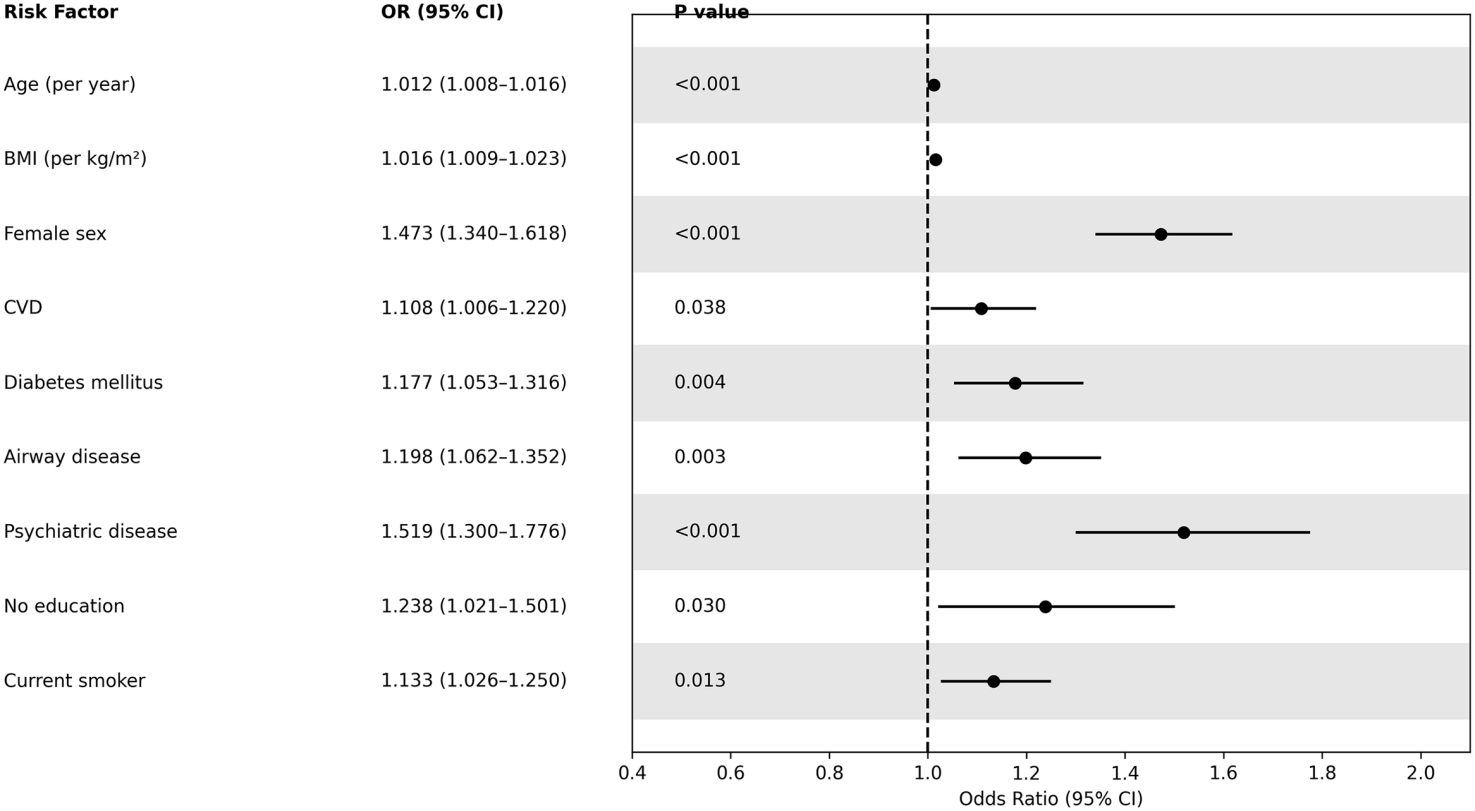
Forest plot showing multivariable-adjusted odds ratios (ORs) and 95% confidence intervals (CIs) for factors independently associated with COMISA. The vertical dashed line indicates no association (OR=1). COMISA: coexistence of insomnia and obstructive sleep apnea.

In multivariable logistic regression analysis adjusted for age, sex, BMI, education level, smoking status, airway disease, diabetes mellitus, and psychiatric comorbidity, CVD remained independently associated with COMISA (odds ratio 1.108, 95% confidence interval 1.006–1.220; p = 0.038). Older age, female sex, higher BMI, lower education level, current smoking, diabetes mellitus, airway disease, and psychiatric comorbidity were also independently associated with COMISA (Fig 3).

The prevalence of individual CVD components according to sleep phenotype is presented in Table 2. Hypertension was the most prevalent component across all groups, whereas coronary artery disease, heart failure, atrial fibrillation, and stroke were consistently more common among participants with COMISA than among the other sleep phenotypes.

**Table 2:** Distribution of individual cardiovascular disease components according to sleep phenotype.

CVD components	Neither insomnia nor OSA	Insomnia only	OSA only	COMISA	p-value
**Hypertension, %**	16.3	23.2	30.5	39.0	<0.001
**CAD, %**	8.8	12.6	14.9	18.6	<0.001
**CHF, %**	1.9	4.4	4.9	7.2	<0.001
**AF, %**	2.2	5.7	3.9	6.3	<0.001
**Stroke, %**	0.3	0.5	0.7	0.9	0.397

Categorical data are presented as percentage Abbreviations: AF: atrial fibrillation; CAD: coronary artery disease; CHF: chronic heart failurex; COMISA: comorbid obstructive sleep apnea and insomnia; CVD: cardiovascular diseases; OSA:obstructive sleep apnea.

Among patients with COMISA, those with CVD had a more adverse clinical and symptomatic profile than those without CVD (Table 3). They were older, had higher BMI, and more frequently had obesity, diabetes mellitus, airway disease, and psychiatric comorbidity (for all p < 0.001). They also reported more sleep-related and daytime symptoms, including nocturia, witnessed apnea, nocturnal dyspnea, night sweats, restless legs syndrome, fatigue, daytime sleepiness, difficulty concentrating, and depressed mood.

**Table 3:** The comparison of demographical and clinical factors in COMISA patients with and without Cardiovascular Diseases (n = 3275).

Variable	CVD (+) (n = 1384)	CVD (-) (n = 1891)	p value
**Age (years) (mean ± SD)**	56.7 ± 10.7	48.6 ± 11.7	<0.001
**BMI (kg/m2) (mean ± SD)**	33.4 ± 6.4	31.7 ± 6.0	<0.001
**Female/ male (n)**	640/744	671/1220	<0.001
**Obesity (BMI>=30 kg/m2) (%)**	67.4	56.4	<0.001
**Current smoker (%)**	26.1	39.5	<0.001
**Married (%)**	83.2	79.7	0.013
**ESS > 10 (%)**	31.7	27.4	0.007
**No education (%)** **Primary school (%)** **Secondary school (%)** **High school (%)** **University (%)**	9.6	7.6	<0.001
	34.1	27.5	
	10.2	10.7	
	25.1	26.6	
	21.0	27.7	
**Difficulty concentrating (%)**	29.3	24.2	0.002
**Depressed mood (%)**	23.8	20.2	0.018
**Nocturia (%)**	39.5	24.5	<0.001
**Witnessed apnea (%)**	47.0	37.2	<0.001
**Snoring (%)**	75.9	69.8	<0.001
**Nocturnal dyspnea (%)**	46.6	36.8	<0.001
**Night sweats (%)**	37.4	25.7	<0.001
**Restless Legs Syndrome (%)**	24.6	18.4	<0.001
**Fatigue (%)**	59.9	46.9	<0.001
**Daytime sleepiness (%)**	56.8	45.2	<0.001
**DM (%)**	41.2	9.3	<0.001
**Airway Disease (%)**	24.2	10.2	<0.001
**Psychiatric Disease (%)**	13.1	6.4	<0.001

**Abbreviations**: BMI: body mass index; CVD: cardiovascular diseases; COMISA: comorbid insomnia and sleep apnea; DM: diabetes mellitus; ESS: Epworth Sleepiness Scale.

Polysomnographic comparisons within the COMISA group showed that patients with CVD had poorer sleep continuity and more severe nocturnal hypoxemia. They had shorter total sleep time, longer wake after sleep onset, lower sleep efficiency, higher AHI and ODI, longer time spent with SpO_2_ < 90%, and lower nadir SpO_2_ (for all p < 0.001). In contrast, neither arousal index nor the proportion of N3 sleep differed significantly between COMISA patients with and without CVD (Table 4).

**Table 4:** Polysomnographic features of COMISA patients with and without Cardiovascular Diseases.

Variable	CVD (+) (n = 1384)	CVD (-) (n = 1891)	P value
**TST (min)**	345.2 ± 79.3	367.0 ± 76.3	<0.001
**WASO (min)**	64.1 ± 50.1	46.2 ± 40.7	<0.001
**Sleep Efficiency (%)**	82.6 ± 13.6	85.4 ± 11.9	<0.001
**N1 (%)**	8.1 ± 9.7	5.5 ± 8.3	<0.001
**N2 (%)**	57.8 ± 17.6	59.3 ± 16.9	0.018
**N3 (%)**	22.6 ± 14.1	23.0 ± 13.9	0.5
**REM (%)**	13.6 ± 8.0	14.2 ± 8.9	0.032
**AHI (events/hr)**	36.1 ± 26.3	32.1 ± 25.3	<0.001
**ODI (events/hr)**	30.8 ± 24.4	27.4 ± 23.8	<0.001
**Time spent SpO**_**2**_ **< 90 (min)**	57.8 ± 89.0	44.5 ± 74.4	<0.001
**Nadir SpO**_**2**_ **(%)**	78.0 ± 11.1	79.4 ± 10.9	<0.001
**Arousal index (events/hr)**	22.9 ± 24.0	22.4 ± 26.8	0.6

Continuous data are presented as mean ± Standard deviation. Categorical data are presented as percentage. **Abbreviations:** AHI: apnea-hypopnea index; CVD: cardiovascular diseases; COMISA: comorbid insomnia and sleep apnea; N1: non-rapid eye movement sleep stage 1; N2: non-rapid eye movement sleep stage 2; N3: non-rapid eye movement sleep stage 3; ODI: oxygen desaturation index; PLM: periodic limb movement; REM: rapid eye movement sleep; SpO_2_: oxygen saturation by pulse oximeter; TST: total sleep time; WASO: wake after sleep onset.

Sensitivity analyses excluding participants classified as having insomnia solely on the basis of hypnotic use resulted in the reclassification of only 113 participants (0.9% of the cohort) (Table 5).

**Table 5:** Distribution of subgroups based on insomnia symptoms including vs excluding individuals on the basis of hypnotic use.

Subgroups	Including hypnotic use	Excluding hypnotic use
**Neither insomnia nor OSA (n)**	910	922
**Insomnia only (n)**	388	376
**OSA only (n)**	8142	8243
**COMISA (n)**	3275	3174

Abbreviations: COMISA: comorbid obstructive sleep apnea and insomnia; OSA:obstructive sleep apnea.

Overall, these findings suggest that COMISA is associated with a higher burden of CVD and that COMISA patients with CVD represent a clinically more vulnerable subgroup characterized by greater cardiometabolic comorbidity, more prominent sleep-related symptoms, poorer sleep continuity, and more severe nocturnal hypoxemia.

## Discussion

In this large nationwide registry-based cohort of 12,715 adults referred for sleep evaluation, COMISA was more frequent among patients with CVD than among those without CVD. Although the adjusted association between COMISA and CVD was modest, it remained statistically significant after adjustment for age, sex, BMI, education level, smoking status, airway disease, diabetes mellitus, and psychiatric comorbidity. In addition, among patients with COMISA, those with CVD showed a more adverse clinical and polysomnographic profile, characterized by older age, higher BMI, greater cardiometabolic and respiratory comorbidity burden, more prominent sleep-related symptoms, poorer sleep continuity, and more severe nocturnal hypoxemia. These findings support the concept that COMISA represents a clinically relevant sleep phenotype associated with increased cardiovascular vulnerability. However, because of the cross-sectional design, these findings should be interpreted as associations rather than evidence of a causal relationship between COMISA and cardiovascular disease.

Our findings are consistent with previous population-based, clinical, and review studies suggesting that COMISA is not merely the coexistence of two common sleep disorders, but rather a distinct clinical phenotype characterized by unique pathophysiological interactions, greater symptom burden, impaired daytime functioning, and increased cardiometabolic vulnerability [1,3–5,8,10,16–19]. In a large propensity score–matched cohort, Lu et al. reported that COMISA was associated with increased long-term risks of cerebrovascular disease, arrhythmias, inflammatory and ischemic heart disease, and thrombotic disorders compared with OSA alone [3]. Similarly, Gaffey et al. showed in a large cohort of U.S. Veterans that COMISA was associated with the greatest risk of incident hypertension and CVD compared with insomnia alone, OSA alone, or no sleep disorder [8]. Our study extends these observations to a large real-world sleep clinic population from Türkiye and demonstrates that COMISA is associated with a higher prevalent CVD burden even after adjustment for major clinical confounders. It is also important to interpret the relatively small insomnia-only group in the context of the high coexistence of insomnia symptoms and OSA in sleep clinic populations. Overall, 3,663 participants (28.8% of the cohort) reported insomnia symptoms, of whom 3,275 (89.4%) also met diagnostic criteria for OSA and were therefore classified as having COMISA. Consequently, only 388 participants were classified as having insomnia symptoms without OSA. This finding further supports the concept that, in sleep clinic populations, insomnia symptoms are far more likely to occur in combination with OSA than as an isolated condition.

The magnitude of the adjusted association in our study was modest. This is clinically important and should be interpreted cautiously. Similarly, the large sample size increased statistical power, and therefore statistical significance should be interpreted together with the magnitude and clinical relevance of the observed associations. COMISA was associated with CVD with an adjusted odds ratio of 1.11, suggesting that its contribution to cardiovascular burden may be relatively small when traditional cardiovascular risk factors and comorbidities are accounted for. This may reflect the multifactorial nature of CVD in sleep clinic populations, where age, obesity, diabetes, smoking, airway disease, and psychiatric comorbidity all contribute substantially to cardiovascular risk. It may also indicate that COMISA is not a single homogeneous phenotype, but includes subgroups with different biological risk profiles. Previous studies have suggested that the cardiovascular risk associated with COMISA may be stronger in patients with objective short sleep duration, severe insomnia symptoms, resistant or uncontrolled hypertension, or more severe nocturnal hypoxemia [9–11,20,21].

Hypertension-related outcomes have been among the most consistently reported cardiovascular associations of COMISA. Previous studies have demonstrated independent associations between COMISA and uncontrolled or resistant hypertension, particularly in individuals with greater nocturnal hypoxemia or objective short sleep duration [4,9,10]. These findings are consistent with our observation that COMISA patients with CVD exhibited more severe nocturnal hypoxemia, reflected by higher AHI and ODI, longer time spent with SpO_2_ < 90%, and lower nadir SpO_2_. Interestingly, despite more severe nocturnal hypoxemia, neither arousal index nor the proportion of N3 sleep differed significantly between COMISA patients with and without CVD. This observation suggests that the severity of nocturnal hypoxemia may be more closely associated with cardiovascular vulnerability than arousal frequency or sleep architecture alone. Nevertheless, given the cross-sectional design, this interpretation should be considered hypothesis-generating and requires confirmation in prospective studies.

Several pathophysiological mechanisms may explain the observed association between COMISA and CVD. OSA contributes to cardiovascular injury through intermittent hypoxemia, sympathetic activation, oxidative stress, endothelial dysfunction, systemic inflammation, intrathoracic pressure swings, and sleep fragmentation [2,3,22,23]. Insomnia is associated with hyperarousal, hypothalamic–pituitary–adrenal axis activation, increased sympathetic tone, inflammation, and impaired metabolic regulation [24,25]. When OSA and insomnia coexist, these mechanisms may converge and amplify cardiometabolic stress. The combination of respiratory-related hypoxemia and insomnia-related hyperarousal may result in persistent autonomic activation, impaired nocturnal blood pressure dipping, increased inflammatory activity, and poorer metabolic control. This model is supported by Pejovic et al., who showed that OSA with insomnia symptoms and objective short sleep duration was associated with higher blood pressure, increased hypertension risk, elevated IL-6 levels, and greater insulin resistance [11]. Interestingly, despite more severe nocturnal hypoxemia, neither arousal index nor N3 sleep differed significantly between COMISA patients with and without CVD. This finding suggests that nocturnal hypoxemia, rather than arousal frequency or sleep architecture alone, may be more closely related to cardiovascular vulnerability in this population.

Our findings also align with studies performed in metabolically or psychiatrically vulnerable populations. Draelants et al. reported that among hypertensive subjects, COMISA—but not OSA or insomnia alone—was independently associated with high 10-year CVD risk assessed by the Framingham Risk Score [6]. Hein et al. found a similar pattern in patients with major depression, where COMISA was associated with high 10-year cardiovascular risk, whereas insomnia disorder or OSA alone were not independently associated with this outcome [12]. In patients with type 2 diabetes, COMISA was also associated with higher cardiovascular risk compared with either sleep disorder alone [13]. These studies suggest that COMISA may exert a stronger adverse cardiovascular effect in individuals with pre-existing metabolic, vascular, or psychiatric vulnerability.

The polysomnographic findings among COMISA patients with CVD provide additional insight. These patients had shorter total sleep time, longer wake after sleep onset, lower sleep efficiency, higher AHI and ODI, longer nocturnal hypoxemia, and lower nadir oxygen saturation. Interestingly, arousal index did not differ significantly between COMISA patients with and without CVD. This may suggest that hypoxemic burden and sleep continuity measures are more closely linked to CVD burden than arousal frequency alone in this cohort. This interpretation is consistent with recent literature suggesting that traditional AHI may not fully capture cardiovascular risk and that oxygen desaturation metrics, hypoxic burden, and sleep duration may provide additional prognostic information [26–28]. Therefore, patients with COMISA may require a more comprehensive sleep evaluation beyond AHI alone, including assessment of nocturnal hypoxemia, sleep duration, and insomnia symptom profile.

From a clinical perspective, these findings emphasize the importance of systematically screening for insomnia symptoms in patients with OSA, particularly in those with cardiovascular or metabolic comorbidities. COMISA may identify a subgroup of patients with higher symptom burden, poorer sleep quality, and greater cardiometabolic vulnerability. Current clinical management of OSA often focuses primarily on respiratory event control, particularly with positive airway pressure therapy. However, randomized cardiovascular outcome trials of CPAP in OSA have produced largely neutral or modest cardiovascular benefits. This may partly reflect the heterogeneity of OSA populations, variable treatment adherence, and the fact that coexisting sleep disturbances such as insomnia are not routinely considered in cardiovascular outcome trials [29–31]. Integrated management strategies that combine OSA treatment with targeted insomnia therapy, such as cognitive behavioral therapy for insomnia, may be necessary to improve both sleep and cardiometabolic outcomes in COMISA [17,32].

The present study has several strengths. It was based on a large nationwide multicenter registry and included more than 12,000 adults referred for sleep evaluation. The use of mutually exclusive sleep disorder phenotypes allowed direct comparison of CVD burden across clinically meaningful groups. In addition, the availability of clinical, comorbidity, and polysomnographic data enabled adjustment for several important confounders and characterization of COMISA patients with and without CVD. These features make the study one of the largest real-world analyses evaluating the association between COMISA and CVD in a sleep clinic population.

Several limitations should also be acknowledged. First, this study represents a cross-sectional analysis of baseline data from the ongoing TURKAPNE registry. Consequently, causal inference cannot be established, and incident cardiovascular outcomes could not be evaluated. Longitudinal follow-up analyses are currently underway and will be reported separately. Second, CVD was based on self-reported physician-diagnosed conditions, which may be subject to misclassification. Third, insomnia was defined using registry-based symptom items rather than formal DSM-5-TR or ICSD-3 diagnostic criteria; therefore, the findings should be interpreted as relating to insomnia symptoms rather than insomnia disorder. Fourth, although the analyses were adjusted for multiple confounders, residual confounding by medication use, physical activity, socioeconomic status, depression severity, sleep medication use, and treatment adherence cannot be excluded. Nevertheless, sensitivity analyses excluding participants classified solely on the basis of hypnotic use produced virtually identical results, suggesting that this potential source of misclassification had minimal impact on the main findings. In addition, cardiovascular disease was based on self-reported physician diagnoses recorded within the registry rather than independent adjudication or review of medical records. Although this approach reflects routine clinical practice, some degree of recall bias or disease misclassification cannot be excluded. Finally, the reference group consisted of individuals referred for sleep evaluation rather than healthy population controls, which may have attenuated differences between sleep phenotypes and may limit generalizability to the general population.

## Conclusion

This large TURKAPNE registry analysis shows that COMISA is more common among patients with CVD and remains independently associated with CVD after adjustment for established clinical risk factors, although the magnitude of the association is modest. Among patients with COMISA, the presence of CVD is associated with a more adverse clinical and polysomnographic profile, including greater obesity burden, higher rates of diabetes and airway disease, more prominent sleep-related symptoms, poorer sleep continuity, and more severe nocturnal hypoxemia. These findings support the clinical relevance of COMISA as a cardiovascular risk phenotype and highlight the need for prospective studies to determine whether integrated treatment of both OSA and insomnia can reduce cardiovascular risk. Future sleep registries should incorporate standardized diagnostic criteria for insomnia disorder, including symptom duration, frequency, daytime impairment, and sleep opportunity, to improve characterization of COMISA phenotypes and their cardiovascular implications.

## Supporting information

S1 File EthicsApprovalTranslation. (PDF)

S2 File HumanParticipantResearchChecklist. (PDF)
