# Supplementary material for: Association between COMISA and cardiovascular disease: Evidence from the TURKAPNE registry
Source: PLoS One. 2026 Jul 31;21(7):e0354810. doi: 10.1371/journal.pone.0354810 (PMC13426967; doi:10.1371/journal.pone.0354810)
Supplement: S1 File — (PDF) [file pone.0354810.s001.pdf]

Unofficial English Translation of Ethics Committee Approval

Marmara University Faculty of Medicine
Clinical Research Ethics Committee

Application Information

Protocol Code: 09.2016.311

Project Title:
TURKAPNE (Turkish Sleep Apnea Database): A national, multicenter, prospective, observational cohort study

Principal Investigator:
Prof. Dr. Yüksel Peker

Decision Information

Date: 13.05.2016

The research application file and related documents listed above were reviewed by the Clinical Research Ethics Committee with respect to the rationale, aims, methodology, and ethical aspects of the study. The committee concluded that there were no ethical objections to conducting the study.

Any modifications to the study protocol, investigators, study title, or study procedures after approval must be reported to the Ethics Committee.
