# Supplementary material for: Association between COMISA and cardiovascular disease: Evidence from the TURKAPNE registry
Source: PLoS One. 2026 Jul 31;21(7):e0354810. doi: 10.1371/journal.pone.0354810 (PMC13426967; doi:10.1371/journal.pone.0354810)
Supplement: Attachment — Submitted filename: Reviewer2.doc [file pone.0354810.s004.doc]

**Reviewer #2:**

*The authors examined the association of COMISA and CVD and published their evidence from TURKAPNE Registry. There are few concerns that warrants attention.*

*1.Although authors acknowledge the cross-sectional nature of the study. This deserves more acknowledgement in the discussion and interpretation.*

-We thank the reviewer for this important comment. We agree that the cross-sectional design deserves greater emphasis when interpreting our findings. Accordingly, we have expanded both the Discussion and the Limitations sections to explicitly state that the observed associations should not be interpreted as causal and that longitudinal analyses from the ongoing TURKAPNE registry will be required to clarify temporal relationships between COMISA and cardiovascular disease.

*2. Insomnia definition is substantiative weakness. Insomnia is defined by self-reported symptoms and does not comprise the ICSD 3 or DSM criteria. This limits comparison with the prior studies which uses more rigorous definition. Furthermore, the use of hypnotics could also misclassify subjects as some patients use hypnotics for other reasons. Consider performing sensitivity analyses restricting the insomnia definition to those with difficulty initiation and maintain sleep.*

- We thank the reviewer for this thoughtful and important comment. We fully agree that the absence of information on symptom frequency, duration, daytime impairment, and adequate sleep opportunity precluded a formal diagnosis of insomnia disorder according to ICSD-3 or DSM-5 criteria. For this reason, we consistently use the term insomnia symptoms throughout the manuscript and explicitly acknowledge this limitation in both the Methods and Discussion sections.

We also appreciate the reviewer's concern regarding the use of hypnotic medication as part of the insomnia definition. To address this issue, we performed the requested sensitivity analysis after excluding participants classified as having insomnia solely on the basis of hypnotic use. This resulted in the reclassification of only 113 participants (0.9% of the entire cohort). These results have now been added as Table 5 and are described in the Results section.

*3. Similarly, CVD was used as self-reported diagnosis. This may introduce recall diagnosis. Furthermore, since CVD is defined as composite of various cardiac conditions, the authors should provide the breakdown of CVD components by sleep phenotypes.*

-We thank the reviewer for this important comment. We agree that the use of self-reported physician-diagnosed cardiovascular disease may have introduced some degree of misclassification or recall bias. We have strengthened the Discussion by explicitly acknowledging this limitation.

To further address the reviewer's suggestion, we have added a new Table 2 presenting the distribution of individual cardiovascular disease components (hypertension, coronary artery disease, heart failure, atrial fibrillation, and stroke) across the four sleep phenotypes.

*4. The observation that arousal index did not differ between the two groups warrant more attention. It suggests that hypoxemia rather than arousal frequency is primary driver of CV vulnerability in COMISA****.***

-We thank the reviewer for this insightful comment. We agree that the absence of differences in arousal index despite significantly more severe nocturnal hypoxemia is an important finding. Accordingly, we have expanded the Discussion to highlight that nocturnal hypoxemia may be more closely associated with cardiovascular vulnerability in COMISA than arousal frequency alone. However, given the cross-sectional design, this interpretation should be considered hypothesis-generating rather than causal.
