# Supplementary material for: Association between COMISA and cardiovascular disease: Evidence from the TURKAPNE registry
Source: PLoS One. 2026 Jul 31;21(7):e0354810. doi: 10.1371/journal.pone.0354810 (PMC13426967; doi:10.1371/journal.pone.0354810)
Supplement: Peer review history

# Peer review: Decision Letter 0

**DOI:** 10.1371/journal.pone.0354810.r001

**Authors:** Jag Sunderram

30 Jun 2026

PONE-D-26-22444

Association Between COMISA and Cardiovascular Disease: Evidence from the TURKAPNE Registry

PLOS One

Dear Dr. Peker,

Thank you for submitting your manuscript to PLOS ONE. After careful consideration, we feel that it has merit but does not fully meet PLOS ONE’s publication criteria as it currently stands. Therefore, we invite you to submit a revised version of the manuscript that addresses the points raised during the review process by each of the reviewers.

Please submit your revised manuscript by Jul 30 2026 11:59PM. If you will need more time than this to complete your revisions, please reply to this message or contact the journal office at plosone@plos.org. When you're ready to submit your revision, log on to https://www.editorialmanager.com/pone/ and select the 'Submissions Needing Revision' folder to locate your manuscript file.

Please include the following items when submitting your revised manuscript:

- A letter that responds to each point raised by the academic editor and reviewer(s). You should upload this letter as a separate file labeled 'Response to Reviewers'.
- A marked-up copy of your manuscript that highlights changes made to the original version. You should upload this as a separate file labeled 'Revised Manuscript with Track Changes'.
- An unmarked version of your revised paper without tracked changes. You should upload this as a separate file labeled 'Manuscript'.

If you would like to make changes to your financial disclosure, please include your updated statement in your cover letter. Guidelines for resubmitting your figure files are available below the reviewer comments at the end of this letter.

If applicable, we recommend that you deposit your laboratory protocols in protocols.io to enhance the reproducibility of your results. Protocols.io assigns your protocol its own identifier (DOI) so that it can be cited independently in the future. For instructions see: https://journals.plos.org/plosone/s/submission-guidelines#loc-laboratory-protocols. Additionally, PLOS ONE offers an option for publishing peer-reviewed Lab Protocol articles, which describe protocols hosted on protocols.io. Read more information on sharing protocols at https://plos.org/protocols?utm_medium=editorial-email&utm_source=authorletters&utm_campaign=protocols.

As the corresponding author, your ORCID iD is verified in the submission system and will appear in the published article. PLOS supports the use of ORCID, and we encourage all coauthors to register for an ORCID iD and use it as well. Please encourage your coauthors to verify their ORCID iD within the submission system before final acceptance, as unverified ORCID iDs will not appear in the published article. *Only*  the individual author can complete the verification step; PLOS staff *cannot*  verify ORCID iDs on behalf of authors.

We look forward to receiving your revised manuscript.

Kind regards,

Jag Sunderram, M.D.

Academic Editor

PLOS One

Journal Requirements:

When submitting your revision, we need you to address these additional requirements.

1. Please ensure that your manuscript meets PLOS ONE's style requirements, including those for file naming. The PLOS ONE style templates can be found at

https://journals.plos.org/plosone/s/file?id=wjVg/PLOSOne_formatting_sample_main_body.pdf and https://journals.plos.org/plosone/s/file?id=ba62/PLOSOne_formatting_sample_title_authors_affiliations.pdf

2. One of the noted authors is a group or consortium [TURKAPNE Study Group]. In addition to naming the author group, please list the individual authors and affiliations within this group in the acknowledgments section of your manuscript. Please also indicate clearly a lead author for this group along with a contact email address.

3. In the online submission form, you indicated that data collected for the study, including de-identified individual participant data will be made available to others within 6 months after the publication of this article, as will additional related documents (study protocol, statistical analysis plan, and informed consent form), for academic purposes (e.g., meta-analyses), upon request to the senior author (yuksel.peker@lungall.gu.se), and with a signed data access agreement.

All PLOS journals now require all data underlying the findings described in their manuscript to be freely available to other researchers, either 1. In a public repository, 2. Within the manuscript itself, or 3. Uploaded as supplementary information.

This policy applies to all data except where public deposition would breach compliance with the protocol approved by your research ethics board. If your data cannot be made publicly available for ethical or legal reasons (e.g., public availability would compromise patient privacy), please explain your reasons on resubmission and your exemption request will be escalated for approval.

4. Thank you for stating the following financial disclosure:

Turkish Thoracic Society

Please state what role the funders took in the study. If the funders had no role, please state: "The funders had no role in study design, data collection and analysis, decision to publish, or preparation of the manuscript."

If this statement is not correct you must amend it as needed.

Please include this amended Role of Funder statement in your cover letter; we will change the online submission form on your behalf.

5. One of the noted authors is a group or consortium [Turkapne Study Group]. In addition to naming the author group, please list the individual authors and affiliations within this group in the acknowledgments section of your manuscript. Please also indicate clearly a lead author for this group along with a contact email address.

6. Please include captions for your Supporting Information files at the end of your manuscript, and update any in-text citations to match accordingly. Please see our Supporting Information guidelines for more information: http://journals.plos.org/plosone/s/supporting-information.

7. If the reviewer comments include a recommendation to cite specific previously published works, please review and evaluate these publications to determine whether they are relevant and should be cited. There is no requirement to cite these works unless the editor has indicated otherwise.

8. Please review your reference list to ensure that it is complete and correct. If you have cited papers that have been retracted, please include the rationale for doing so in the manuscript text, or remove these references and replace them with relevant current references. Any changes to the reference list should be mentioned in the rebuttal letter that accompanies your revised manuscript. If you need to cite a retracted article, indicate the article’s retracted status in the References list and also include a citation and full reference for the retraction notice.

Additional Editor Comments :

Please address concerns of both reviewers and send us the revised manuscript.

[Note: HTML markup is below. Please do not edit.]

Reviewers' comments:

Reviewer's Responses to Questions

**Comments to the Author**

1. Is the manuscript technically sound, and do the data support the conclusions?

The manuscript must describe a technically sound piece of scientific research with data that supports the conclusions. Experiments must have been conducted rigorously, with appropriate controls, replication, and sample sizes. The conclusions must be drawn appropriately based on the data presented.

Reviewer #1: Yes

Reviewer #2: Yes

**********

2. Has the statistical analysis been performed appropriately and rigorously?

Reviewer #1: Yes

Reviewer #2: I Don't Know

**********

3. Have the authors made all data underlying the findings in their manuscript fully available?

The PLOS Data policy requires authors to make all data underlying the findings described in their manuscript fully available without restriction, with rare exception (please refer to the Data Availability Statement in the manuscript PDF file). The data should be provided as part of the manuscript or its supporting information, or deposited to a public repository. For example, in addition to summary statistics, the data points behind means, medians and variance measures should be available. If there are restrictions on publicly sharing data—e.g. participant privacy or use of data from a third party—those must be specified.

Reviewer #1: Yes

Reviewer #2: Yes

**********

4. Is the manuscript presented in an intelligible fashion and written in standard English?

PLOS ONE does not copyedit accepted manuscripts, so the language in submitted articles must be clear, correct, and unambiguous. Any typographical or grammatical errors should be corrected at revision, so please note any specific errors here.

Reviewer #1: Yes

Reviewer #2: Yes

**********

5. Review Comments to the Author

Please use the space provided to explain your answers to the questions above. You may also include additional comments for the author, including concerns about dual publication, research ethics, or publication ethics. (Please upload your review as an attachment if it exceeds 20,000 characters)

Reviewer #1: The paper "Association Between COMISA and Cardiovascular Disease: Evidence from the TURKAPNE Registry" is an original analysis on a real-life large cohort of OSA and insomnia patients with solid statistical analysis and good evidence with clinical significance. Therea re some issues that can be solved.

Methods, study population. Do you have some follow-up data of the patients form your cohort?

Line 206. Insomnia symptoms rather than insomnia disorder. You mentioned it tin the limitation chapter. 388 patients from 12715 of total number. This is a rather low incidence, in a population refer to a sleep lab. Can you comment? Probably all cohorts should focus now on getting specific insomnia data to define insomnia disorder.

Line 245. Table 1. All values are significant different!

Line 251. You mentioned male genre as independently associated with CVD. But females have OR 1.47. figure 3. Can you comment?

Line 259. Nocturnal hypoxemic burden. Well define in the paper, because it can be confused with the hypoxic burden, explained in the discussion chapter.

Line 261. "In contrast, arousal index did not differ significantly between COMISA patients with and without CVD (Table 3). " N3, also. p.05.

Discussion. A bit long, with some repetitive ideas from similar references, as mentioned in the introduction. But I think it fits within the journal recommendation. You mentioned the modest effect size and the multiple behavioural and metabolic influences.

Conclusion. For practical reasons, we must collect more specific insomnia data in all sleep cohorts, due to significant association and clinical consequences.

Reviewer #2: The authors examined the association of COMISA and CVD and published their evidence from TURKAPNE Registry. There are few concerns that warrants attention.

1. Although authors acknowledge the cross-sectional nature of the study. This deserves more acknowledgement in the discussion and interpretation.

2. Insomnia definition is substantiative weakness. Insomnia is defined by self-reported symptoms and does not comprise the ICSD 3 or DSM criteria. This limits comparison with the prior studies which uses more rigorous definition. Furthermore, the use of hypnotics could also misclassify subjects as some patients use hypnotics for other reasons. Consider performing sensitivity analyses restricting the insomnia definition to those with difficulty initiation and maintain sleep.

3. Similarly, CVD was used as self-reported diagnosis. This may introduce recall diagnosis. Furthermore, since CVD is defined as composite of various cardiac conditions, the authors should provide the breakdown of CVD components by sleep phenotypes.

4. The observation that arousal index did not differ between the two groups warrant more attention. it suggests that hypoxemia rather than arousal frequency is primary driver of CV vulnerability in COMISA.

**********

6. PLOS authors have the option to publish the peer review history of their article (what does this mean?). If published, this will include your full peer review and any attached files.

If you choose “no”, your identity will remain anonymous but your review may still be made public.

**Do you want your identity to be public for this peer review?**  For information about this choice, including consent withdrawal, please see our Privacy Policy.

Reviewer #1: **Yes:**  Stefan Mihaicuta

Reviewer #2: No

**********

[NOTE: If reviewer comments were submitted as an attachment file, they will be attached to this email and accessible via the submission site. Please log into your account, locate the manuscript record, and check for the action link "View Attachments". If this link does not appear, there are no attachment files.]

To ensure your figures meet our technical requirements, please review our figure guidelines: https://journals.plos.org/plosone/s/figures

You may also use PLOS’s free figure tool, NAAS, to help you prepare publication quality figures: https://journals.plos.org/plosone/s/figures#loc-tools-for-figure-preparation.

NAAS will assess whether your figures meet our technical requirements by comparing each figure against our figure specifications.

# Author comment: Author response to Decision Letter 1

**DOI:** 10.1371/journal.pone.0354810.r002

2 Jul 2026

Reviewer #1:

The paper "Association Between COMISA and Cardiovascular Disease: Evidence from the TURKAPNE Registry" is an original analysis on a real-life large cohort of OSA and insomnia patients with solid statistical analysis and good evidence with clinical significance. There are some issues that can be solved.

Methods, study population. Do you have some follow-up data of the patients form your cohort?

- We thank the reviewer for this important comment. The present study was designed as a cross-sectional analysis of the baseline TURKAPNE registry and therefore does not include longitudinal follow-up data. However, the TURKAPNE registry is an ongoing prospective nationwide cohort, and longitudinal follow-up analyses addressing clinical outcomes are currently underway and will be reported separately in future publications. These clarifications are now added to the Discussion section.

Line 206. Insomnia symptoms rather than insomnia disorder. You mentioned it in the limitation chapter. 388 patients from 12715 of total number. This is a rather low incidence, in a population refer to a sleep lab. Can you comment? Probably all cohorts should focus now on getting specific insomnia data to define insomnia disorder.

-We thank the reviewer for this thoughtful observation. We agree that the number of participants classified as having insomnia alone (n=388) appears relatively small in a sleep clinic population. However, this reflects the high coexistence of insomnia symptoms and OSA in our cohort rather than a low prevalence of insomnia symptoms. Overall, 3,663 participants (28.8% of the cohort) reported insomnia symptoms, of whom 3,275 (89.4%) also had OSA and were therefore classified as having COMISA, leaving only 388 participants in the insomnia-only group. This distribution is consistent with previous reports showing that insomnia symptoms frequently coexist with OSA in sleep clinic populations. We have clarified this point in the Discussion.

Line 245. Table 1. All values are significant different!

-We thank the reviewer for this observation. We agree that many variables reached statistical significance. This is most likely attributable to the large sample size of the TURKAPNE cohort (n=12,715), which provided high statistical power to detect even relatively small between-group differences. To facilitate interpretation, we have focused our discussion on findings considered clinically meaningful rather than statistical significance alone.

Line 251. You mentioned male genre as independently associated with CVD. But females have OR 1.47. figure 3. Can you comment?

-We thank the reviewer for identifying this inconsistency. The reviewer is correct. This was an inadvertent wording error in the Results section. The text has been corrected to indicate that female sex, rather than male sex, was independently associated with COMISA, consistent with the multivariable logistic regression analysis presented in Figure 3.

Line 259. Nocturnal hypoxemic burden. Well define in the paper, because it can be confused with the hypoxic burden, explained in the discussion chapter.

-We thank the reviewer for this helpful observation. We agree that the term "nocturnal hypoxemic burden" may be confused with the established metric "hypoxic burden." To avoid this potential ambiguity, we have replaced the term throughout the manuscript with "more severe nocturnal hypoxemia" where appropriate. This terminology more accurately reflects the polysomnographic measures evaluated in our study, including time spent with oxygen saturation <90% and nadir SpO₂.

Line 261. "In contrast, arousal index did not differ significantly between COMISA patients with and without CVD (Table 3). " N3, also. p.05.

-We thank the reviewer for this helpful observation. We agree that the absence of significant differences applied to both arousal index and N3 sleep. Accordingly, we have revised the Results section to state that neither arousal index nor the proportion of N3 sleep differed significantly between COMISA patients with and without CVD. In addition, we have expanded the Discussion to note that the lack of differences in arousal index and N3 sleep, despite more severe nocturnal hypoxemia, suggests that nocturnal hypoxemia may be more closely related to cardiovascular vulnerability in COMISA than arousal frequency or sleep architecture alone.

Discussion. A bit long, with some repetitive ideas from similar references, as mentioned in the introduction. But I think it fits within the journal recommendation. You mentioned the modest effect size and the multiple behavioural and metabolic influences.

-We thank the reviewer for this constructive suggestion. We have revised the Discussion to improve its conciseness by reducing repetitive descriptions of previous studies, particularly in the section addressing hypertension-related findings, while preserving the main scientific message and interpretation of our results.

Conclusion. For practical reasons, we must collect more specific insomnia data in all sleep cohorts, due to significant association and clinical consequences.

-We thank the reviewer for this valuable suggestion. We agree that more comprehensive and standardized assessment of insomnia would improve the characterization of COMISA in future sleep cohorts. Accordingly, we have expanded the Conclusion to emphasize the importance of systematically collecting insomnia-specific data using established diagnostic criteria in future registry studies.

Reviewer #2:

The authors examined the association of COMISA and CVD and published their evidence from TURKAPNE Registry. There are few concerns that warrants attention.

1.Although authors acknowledge the cross-sectional nature of the study. This deserves more acknowledgement in the discussion and interpretation.

-We thank the reviewer for this important comment. We agree that the cross-sectional design deserves greater emphasis when interpreting our findings. Accordingly, we have expanded both the Discussion and the Limitations sections to explicitly state that the observed associations should not be interpreted as causal and that longitudinal analyses from the ongoing TURKAPNE registry will be required to clarify temporal relationships between COMISA and cardiovascular disease.

2. Insomnia definition is substantiative weakness. Insomnia is defined by self-reported symptoms and does not comprise the ICSD 3 or DSM criteria. This limits comparison with the prior studies which uses more rigorous definition. Furthermore, the use of hypnotics could also misclassify subjects as some patients use hypnotics for other reasons. Consider performing sensitivity analyses restricting the insomnia definition to those with difficulty initiation and maintain sleep.

- We thank the reviewer for this thoughtful and important comment. We fully agree that the absence of information on symptom frequency, duration, daytime impairment, and adequate sleep opportunity precluded a formal diagnosis of insomnia disorder according to ICSD-3 or DSM-5 criteria. For this reason, we consistently use the term insomnia symptoms throughout the manuscript and explicitly acknowledge this limitation in both the Methods and Discussion sections.

We also appreciate the reviewer's concern regarding the use of hypnotic medication as part of the insomnia definition. To address this issue, we performed the requested sensitivity analysis after excluding participants classified as having insomnia solely on the basis of hypnotic use. This resulted in the reclassification of only 113 participants (0.9% of the entire cohort). These results have now been added as Table 5 and are described in the Results section.

3. Similarly, CVD was used as self-reported diagnosis. This may introduce recall diagnosis. Furthermore, since CVD is defined as composite of various cardiac conditions, the authors should provide the breakdown of CVD components by sleep phenotypes.

-We thank the reviewer for this important comment. We agree that the use of self-reported physician-diagnosed cardiovascular disease may have introduced some degree of misclassification or recall bias. We have strengthened the Discussion by explicitly acknowledging this limitation.

To further address the reviewer's suggestion, we have added a new Table 2 presenting the distribution of individual cardiovascular disease components (hypertension, coronary artery disease, heart failure, atrial fibrillation, and stroke) across the four sleep phenotypes.

4. The observation that arousal index did not differ between the two groups warrant more attention. It suggests that hypoxemia rather than arousal frequency is primary driver of CV vulnerability in COMISA.

-We thank the reviewer for this insightful comment. We agree that the absence of differences in arousal index despite significantly more severe nocturnal hypoxemia is an important finding. Accordingly, we have expanded the Discussion to highlight that nocturnal hypoxemia may be more closely associated with cardiovascular vulnerability in COMISA than arousal frequency alone. However, given the cross-sectional design, this interpretation should be considered hypothesis-generating rather than causal.

Attachment Submitted filename: Reviewer2.doc

# Editor report: Decision Letter 1

**DOI:** 10.1371/journal.pone.0354810.r003

**Authors:** Jag Sunderram

14 Jul 2026

Association Between COMISA and Cardiovascular Disease: Evidence from the TURKAPNE Registry

PONE-D-26-22444R1

Dear Dr. Peker,

We’re pleased to inform you that your manuscript has been judged scientifically suitable for publication and will be formally accepted for publication once it meets all outstanding technical requirements.

Within one week, you’ll receive an e-mail detailing the required amendments. When these have been addressed, you’ll receive a formal acceptance letter and your manuscript will be scheduled for publication.

An invoice will be generated when your article is formally accepted. Please note, if your institution has a publishing partnership with PLOS and your article meets the relevant criteria, all or part of your publication costs will be covered. Please make sure your user information is up-to-date by logging into Editorial Manager at Editorial Manager® and clicking the ‘Update My Information' link at the top of the page. For questions related to billing, please contact billing support.

If your institution or institutions have a press office, please notify them about your upcoming paper to help maximize its impact. If they’ll be preparing press materials, please inform our press team as soon as possible -- no later than 48 hours after receiving the formal acceptance. Your manuscript will remain under strict press embargo until 2 pm Eastern Time on the date of publication. For more information, please contact onepress@plos.org.

Kind regards,

Jag Sunderram, M.D.

Academic Editor

PLOS One

Additional Editor Comments (optional):

Reviewers' comments:

# Editor report: Acceptance letter

**DOI:** 10.1371/journal.pone.0354810.r004

**Authors:** Jag Sunderram

PONE-D-26-22444R1

PLOS One

Dear Dr. Peker,

I'm pleased to inform you that your manuscript has been deemed suitable for publication in PLOS One. Congratulations! Your manuscript is now being handed over to our production team.

At this stage, our production department will prepare your paper for publication. This includes ensuring the following:

* All references, tables, and figures are properly cited

* All relevant supporting information is included in the manuscript submission,

* There are no issues that prevent the paper from being properly typeset

You will receive further instructions from the production team, including instructions on how to review your proof when it is ready. Please keep in mind that we are working through a large volume of accepted articles, so please give us a few days to review your paper and let you know the next and final steps.

Lastly, if your institution or institutions have a press office, please let them know about your upcoming paper now to help maximize its impact. If they'll be preparing press materials, please inform our press team within the next 48 hours. Your manuscript will remain under strict press embargo until 2 pm Eastern Time on the date of publication. For more information, please contact onepress@plos.org.

You will receive an invoice from PLOS for your publication fee after your manuscript has reached the completed accept phase. If you receive an email requesting payment before acceptance or for any other service, this may be a phishing scheme. Learn how to identify phishing emails and protect your accounts at https://explore.plos.org/phishing.

If we can help with anything else, please email us at customercare@plos.org.

Thank you for submitting your work to PLOS ONE and supporting open access.

Kind regards,

PLOS ONE Editorial Office Staff

on behalf of

Dr. Jag Sunderram

Academic Editor

PLOS One
